# Electron Correlation in Chemical Bonding: From Dispersion to Covalent Stabilization in Prototype Diatomic Systems

**DOI:** 10.1021/acs.jpca.6c02311

**Published:** 2026-09-25

**Authors:** Guo-Qiang Hai, Ladir Cândido, Braulio Gabriel Alencar Brito

**Affiliations:** † Instituto de Física de São Carlos, 117186 Universidade de São Paulo, 13560-970 São Carlos, SP, Brazil; ‡ 67824 Instituto de Física, Universidade Federal de Goiás, 74001-970 Goiânia, GO, Brazil; § Departamento de Física, 74348 Universidade Federal do Triângulo Mineiro, 38025-180 Uberaba, MG, Brazil

## Abstract

The interplay between electron correlation and chemical bonding remains a central issue in quantum chemistry. While covalent bonds arise from electron sharing and long-range interactions from weak correlation, the quantitative connection between these regimes is not fully understood. In this work, we combine ab initio Quantum Monte Carlo (QMC) and Hartree–Fock (HF) calculations to examine how correlation contributes to bonding in the prototypical diatomic systems H_2_, LiH, and Li_2_. By mapping correlation energies and reaction forces along the full dissociation coordinate, we show that correlation gives rise to the asymptotic long-range dispersion attraction between separated neutral fragments, while also modifying the interaction at shorter bond distances. Near equilibrium, HF interactions dominate in H_2_ and LiH, although correlation contributes significantly to the binding energy, reaching about 41% in LiH. In H_2_, the correlation contribution to the interatomic force becomes repulsive near equilibrium, giving a force-level interpretation of the well-known shift of the correlated equilibrium bond length relative to the HF result. In contrast, in Li_2_, correlation accounts for nearly 80% of the binding energy, indicating that stabilization can arise primarily from correlation when HF attraction is weak. These results provide a quantitative and physically transparent characterization of how electron correlation contributes across different bonding regimes in representative diatomic systems. The present analysis offers a consistent interpretation of the evolution from long-range dispersion to short-range covalent stabilization within computationally reliable prototype molecules.

## Graphical abstract

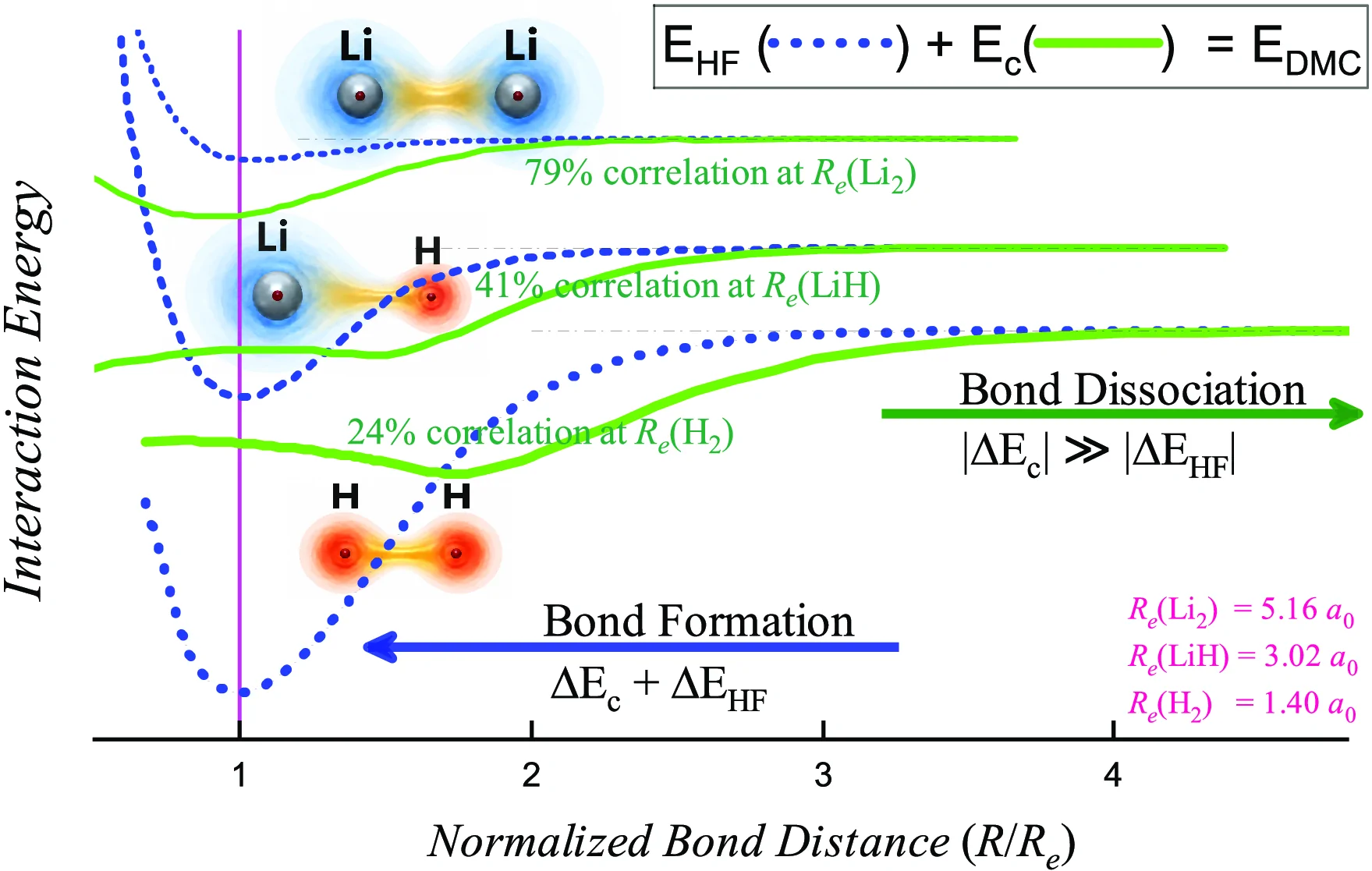

## I Introduction

The nature of the chemical bond remains a central topic in quantum chemistry, with its interpretation evolving significantly since the early electron-pair model of Lewis.^1^ While quantum mechanics provides the formal framework for describing bonding,^2^ the relative roles of kinetic, potential, and correlation energies continue to be actively discussed.^3–9^ In particular, electron correlation plays a dual role in chemical interactions: at large interatomic separations it gives rise to long-range correlation-induced attraction, analogous to dispersion, while at shorter distances it modifies and stabilizes the mean-field description of covalent bonding.^10–13^ Establishing a clear and quantitative connection between these regimes in molecular systems remains an important challenge. The analysis of interatomic forces, particularly through the concept of the reaction force, has also been widely used to characterize bonding and chemical processes.

In this work, we investigate this connection using three prototypical diatomic systems—H_2_, LiH, and Li_2_—chosen because they enable highly accurate Quantum Monte Carlo (QMC) calculations while spanning distinct bonding regimes (covalent, polar, and weakly bound). We focus on H_2_, LiH, and Li_2_ as minimal all-electron diatomic test cases rather than as universal representatives of chemical bonding. H_2_ provides the simplest homonuclear covalent bond, LiH introduces a heteronuclear polar bond with substantial ionic character, and Li_2_ represents a weak, diffuse bond that may also be viewed as a diatomic precursor to metallic bonding in lithium. These systems allow accurate QMC calculations along a single dissociation coordinate and make it possible to separate Hartree–Fock and correlation contributions to both energies and reaction forces in a particularly transparent way. More chemically complex bonds, such as the C–H bond in CH_4_, are important future targets, but they introduce many-center and angular effects that lie beyond the minimal diatomic analysis pursued here. A key aspect of the present work is the explicit decomposition of both total energies and interatomic forces into Hartree–Fock and correlation contributions, enabling a direct identification of correlation-driven regimes. This approach allows a physically transparent decomposition of bonding contributions in these systems and enables identification of crossover regimes and correlation-driven stabilization effects.

Two complementary viewpoints have historically shaped the understanding of chemical bonding. The electrostatic interpretation, developed by Slater, Feynman, and Coulson,^14–16^ and later elaborated within Bader’s Atoms-in-Molecules framework,^17^ emphasizes stabilization arising from the attractive potential energy between the nuclei and the electron density accumulated in the bonding region. More recent developments within Quantum Chemical Topology (QCT), particularly the Interacting Quantum Atoms (IQA) approach, have extended Bader’s real-space perspective by decomposing molecular energies into intra-atomic and interatomic contributions, including exchange and correlation terms. These studies have shown that electron correlation can be analyzed within atoms and between bonded or nonbonded atoms, and that correlation effects may contribute differently depending on bond type, polarity, and dispersion character.^18–21^ The present QMC–UHF analysis is complementary to these QCT/IQA approaches: rather than partitioning the correlated two-particle density matrix in real space, we follow the total Hartree–Fock and correlation contributions to interaction energies and reaction forces along the dissociation coordinate. The quantum-mechanical, or delocalization-based, interpretation, first proposed by Hellmann^22^ and rigorously formulated by Ruedenberg,^3^ attributes covalent bond formation to electron delocalization and the associated changes in kinetic and potential energies, which lower the total energy relative to that of the separated atoms. This mechanism is already present at the HF level; the correlation contribution analyzed in the present work represents the additional energy contribution and force modification beyond the UHF mean-field reference. Ruedenberg’s analysis of H_2_ further revealed kinetic-energy lowering as a key mechanism of covalent bonding.^3,23,24^ Subsequent analyses showed that in heavier diatomics the kinetic energy may increase upon bonding,^4^ while Frenking^5^ argued that quantum interference, rather than electron pairing, underlies stabilization

Modern studies by Bacskay et al.,^23,25,26^ Ruedenberg,^24^ Levine and Head-Gordon,^4^ and Frenking^5^ reconciled electrostatic and correlation effects within a unified variational framework, forming the conceptual basis for the present quantitative examination of Hartree–Fock and correlation interactions derived from Quantum Monte Carlo and unrestricted Hartree–Fock (UHF) calculations. Parallel developments identified dispersion forces as essential in supramolecular systems.^27^ Considerable insight into the connection between long-range dispersion and shorter-range interactions has also been obtained from dispersion-damping models and related density-functional approaches, including the exchange-hole dipole-moment model of Becke and Johnson and subsequent dispersion-corrected methods.^28^ The present work provides a complementary QMC-based analysis by following the residual correlation contribution to energies and forces continuously along molecular dissociation curves. This conceptual gap remains partially unresolved in modern computational chemistry, where density functional theory (DFT) often treats dispersion as an empirical correction,^29^ despite its quantum-mechanical origin.

The central issue concerns the dual role of electron correlation. At all bond distances, electron correlation represents the part of the many-electron interaction missing from the Hartree–Fock mean-field description.^30^ At large separations, this missing contribution gives rise to the long-range dispersion interaction, whereas at shorter bonding distances it includes residual dynamical and short-range correlation effects beyond the UHF reference. Seminal studies by Kołos and Wolniewicz^31^ and Rassolov, Ratner, and Pople^32^ quantified correlation effects in H_2_, while Wetthasinghe and Rassolov^33^ later extended this analysis to LiH. However, these investigations focused on individual molecules over limited ranges of bond distance without uncovering general trends. Fundamental questions therefore remain: How does correlation mediate the transition from weak dispersion to covalent bonding? What determines its relative contribution across different types of bonds?

In this work, we use established fixed-node diffusion Monte Carlo (DMC) methods,^34,35^ together with optimized Slater–Jastrow trial wave functions and present computational resources, to trace correlation energies along the full bond dissociation curves of H_2_, LiH, and Li_2_. These molecules are minimal model systems that isolate the essential physics of correlation-driven attraction and bond stabilization, without the complexities of multishell atoms. They thus serve as reference prototypes for analyzing how correlation effects evolve with bond distance. By comparing with unrestricted Hartree–Fock (UHF) calculations,^30,36^ we demonstrate that correlation contributes up to 79% of Li_2_’s binding energy. This shows that the relative importance of correlation is not determined solely by the absolute bond strength: a weakly bound molecule such as Li_2_ can nevertheless be dominated by the residual correlation contribution beyond the UHF reference. Our analysis further reveals that the correlation contribution to the interatomic force is not uniformly attractive: it is attractive at large separations, where it enhances binding relative to the HF reference, but becomes repulsive near equilibrium in H_2_, shifting the equilibrium distance to larger values. This sign change of the correlation force *F*_c_, from attractive at larger *R* to repulsive near the H_2_ equilibrium region, provides a force-level interpretation of the well-known shift of the correlated equilibrium bond length relative to the HF result. Our results provide a consistent computational framework for quantitatively analyzing how electron correlation modifies the Hartree–Fock interaction from the dispersive to the covalent regime. These insights help clarify connections between different bonding interpretations while delivering benchmarks for next-generation electronic-structure methods.^13^

The aim of this work is not to reformulate bonding theory, but to place these established concepts on a quantitative footing by explicitly separating the Hartree–Fock and correlation interactions along representative diatomic potential curves. The present study highlights the central role of electron correlation in shaping different bonding interactions in these systems—a vision foreshadowed by Löwdin^10,11^ and here quantitatively illustrated across the full spectrum of bonding regimes. In this way, our QMC–UHF results complement the previous conceptual analyses^3–9,23,25,26^ by providing direct numerical evidence of how correlation modifies the mean-field interaction from the dispersive to the covalent regime.

## II Theoretical Framework and Computational Details

The primary objective of this study is to determine highly accurate correlation energies for the diatomic molecules H_2_, LiH, and Li_2_ as a function of bond distance. We compute the ground-state energies of these molecules using fixed-node diffusion Monte Carlo (DMC) simulations,^34,35,37,38^ from bond distances smaller than the equilibrium distance *R*_*e*_ to complete dissociation. These DMC calculations yield total ground-state energies (*E*_DMC_^tot^) with very small statistical errors. For example, the errors are less than 0.05 m*E*_h_ for H_2_ and 0.2 m*E*_h_ for Li_2_. Given that chemical accuracy is conventionally defined as 4.184 kJ/mol (≃ 1.59 m*E*_h_), our results achieve a precision that is much smaller than this threshold. Thus, the ground-state energies from DMC calculations for these systems may successfully capture the fundamental physical and chemical features of molecular formation that are of interest. The final DMC energies used in the present analysis were obtained from production calculations aimed at submilli-Hartree accuracy. The quoted uncertainties include the statistical uncertainty of the DMC averages and the residual effects of time-step and population-control tests. For H_2_, comparison with highly accurate benchmark data shows deviations smaller than 0.1 m*E*_h_. For LiH and Li_2_, the residual uncertainty is estimated to be smaller than 0.4 m*E*_h_.

To quantify the correlation energy, we compare the DMC results with those from a mean-field reference calculation. Unrestricted Hartree–Fock calculations are performed to obtain the total energies (*E*_HF_^tot^) of the molecules without explicit electron correlation beyond the mean-field exchange description. The total correlation energy (*E*_c_^tot^) is then calculated as the difference *E*_DMC_^tot^ – *E*_HF_^tot^ = *E*_c_^tot^. In this work, the correlation energy is defined with respect to the unrestricted Hartree–Fock reference. This corresponds to the Pople-type definition, in which the Hartree–Fock reference is the lowest-energy single-determinant solution, rather than necessarily the restricted Hartree–Fock solution.^12^ Thus, throughout this paper, *E*_c_ denotes *E*_c_ = *E*_DMC_ – *E*_UHF_. This definition differs from the conventional RHF-reference correlation energy, but is useful for bond dissociation because UHF removes much of the artificial RHF dissociation error by allowing spin-symmetry breaking and partial recovery of static left–right correlation. The remaining DMC–UHF difference, therefore, measures the residual correlation beyond the broken-symmetry mean-field description. The same UHF-reference definition was also used in our recent analysis of valence-electron correlation energies in atoms.^39^

We also note that a spin-adapted valence-bond or multiconfigurational reference would provide a more complete description of static correlation, especially for stretched homonuclear molecules such as H_2_ and Li_2_. In the dissociation limit, the UHF solution corresponds to one of two degenerate broken-symmetry determinants, whereas a valence-bond description restores the singlet symmetry through a combination of covalent structures. Such a reference would transfer part of the static correlation currently included in the DMC–UHF difference into the reference energy itself. In the present work, however, we use UHF as a simple and consistent broken-symmetry reference for all three systems so that *E*_c_ should be interpreted as the residual correlation beyond this UHF mean-field description.

From the perspective of dynamical and static correlation, Hollett and Gill^40^ pointed out that the UHF energy can capture part of the static correlation energy and, consequently, the correlation energy defined as the difference between the exact nonrelativistic ground-state energy and the UHF energy is a more robust measure.^41^ The mechanism is the variational relaxation of the spin restriction imposed in RHF. In RHF, the opposite-spin electrons are constrained to occupy the same spatial orbital, whereas in UHF, the α- and β-spin orbitals are optimized independently. In near-degenerate situations, such as stretched H_2_, the bonding σ_*g*_ and antibonding σ_*u*_ orbitals approach degeneracy, and UHF can mix these orbitals differently in the two spin channels. This produces a broken-symmetry determinant in which the two opposite-spin electrons become localized on different atoms, reducing the artificial ionic contribution present in the restricted determinant. In this way, UHF partially mimics the left–right, or near-degeneracy, static correlation, although at the price of spin contamination. As emphasized by Hollett and Gill, this recovery applies mainly to the so-called Type A static correlation, associated with an absolute near-degeneracy, whereas other forms of static correlation are not necessarily recovered by UHF. Their analysis indicates that in stretched H_2_ molecules, the correlation energy within such a definition is dominated by the dynamical effect.

To obtain highly accurate ground-state energies of these molecules, we started with density functional theory (DFT) calculations within an all-electron treatment. We employ the SVWN semilocal exchange–correlation functional and the 6–311+G­(3df) basis set, as implemented in the GAUSSIAN09 program.^42^ For each system, we systematically vary the bond distance in increments of 0.2 *a*_0_, starting from 0.2 *a*_0_ up to 15.0 *a*_0_ for H_2_ and LiH, and up to 20.0 *a*_0_ for Li_2_, ensuring well-converged results across these ranges. At each bond distance, we extract the corresponding molecular orbitals, which are then used in variational Monte Carlo (VMC) calculations to refine the electronic-structure description.

The extracted orbitals serve as input for QMC calculations, which are performed in two steps: VMC for trial wave function optimization, followed by DMC to improve accuracy. The all-electron QMC calculations are performed using the CASINO code.^43^ First, we apply VMC with a Fermionic trial wave function of the Slater-Jastrow form as

1 $$\Psi_{T}\;\left(R\right)=D_{↑}\left(\{\phi_{i}\}\right)D_{↓}\left(\{\phi_{i}\}\right)\;e\mathrm{xp}\;\left[\sum_{I,i<j}U\;\left(r_{\mathrm{iI}},r_{\mathrm{jI}},r_{\mathrm{ij}}\right)\right]$$

where R represents the electronic configuration, *D*_↑_ and *D*_↓_ are the Slater determinants for up- and down-spin orbitals, respectively, and ϕ_*i*_ denotes the one-particle orbitals extracted from the DFT calculations. The Gaussian orbitals used in the Slater determinants are modified using the Ma–Towler–Drummond–Needs scheme to restore the exact electron–nucleus cusp condition,^44^ eliminating divergences in the local energy and improving the nodal quality near the core. For the Jastrow factor *U*, we use the form developed by Drummond, Towler, and Needs,^45^ which is expanded in a suitable basis set to incorporate electron–electron, electron–core, and electron–core–electron correlation terms: *u*(*r*_*ij*_, α), χ­(*r*_*iI*_, β), and *f*(*r*_*iI*_, *r*_*jI*_, *r*_*ij*_, η), respectively. Here, *i*, *j* denote electrons, *I* denotes atomic cores, and *r*_*iI*_, *r*_*jI*_, and *r*_*ij*_ are the corresponding distances. The parameters α, β, and η are optimized by minimizing the variance of the VMC energy.^46^ We also note that compact explicitly correlated wave functions, such as those discussed by Turbiner et al.^47^ for few-electron Coulomb systems, can reach benchmark-level accuracy for selected states; the present DMC calculations pursue a complementary goal, namely, a consistent treatment of complete molecular dissociation curves and the corresponding force decomposition.

Next, we perform DMC simulations to reduce the variational bias. In this method, the imaginary time evolution operator e^–*τH*^ is applied iteratively within the short-time approximation, allowing the trial wave function to evolve toward the ground state in the long-time limit (τ → ∞) by eliminating higher excited states.^34,48^ We employ the fixed-node DMC approximation, in which the nodal surfaces of the wave function are constrained to those of the trial wave function,^35^ introducing a variational upper bound on the ground-state energy. All fixed-node DMC calculations employ a single-determinant Slater–Jastrow form. For small diatomic molecules, this form reproduces total energies within 1–2 m*E*_h_ of multideterminant benchmarks,^45^ ensuring that the qualitative and quantitative trends in the correlation energy and reaction forces are unaffected by residual nodal errors. To assess nodal dependence, FN-DMC energies were recalculated using orbitals generated by both the SVWN and PBE functionals. The resulting total energies differed by less than 0.2 m*E*_h_, indicating that the nodal surfaces are insensitive to the specific mean-field functional for these small diatomic molecules.

For the few-electron systems considered here, full configuration interaction within a finite Gaussian basis is, in principle, feasible and provides valuable benchmark data. The purpose of using VMC/DMC in the present work is different: we use QMC as a uniform real-space correlated reference for H_2_, LiH, and Li_2_, allowing the interaction energies and reaction forces to be analyzed consistently along the complete dissociation coordinates. The optimized Jastrow factor explicitly accounts for electron–electron correlation and cusp behavior, thereby improving the quality and stability of the trial wave functions used in VMC/DMC.

To ensure the accuracy of the DMC calculations, we perform test calculations with varying time steps (0.0005–0.0001au) and linearly extrapolate the energies to zero time step for all studied molecules. At sufficiently small time steps, the DMC time step bias scales approximately linearly with Δ*τ* due to first-order errors in the short-time propagator. Conversely, the bias associated with the finite walker population is inversely proportional to the number of walkers, as statistical fluctuations decrease with larger ensemble sizes.^49^ By applying a linear extrapolation to zero time step, we effectively reduce both time step and population-control biases, as discussed in ref^50^. This approach minimizes discretization errors associated with finite time steps in DMC.

From these tests, we find that using a time step of 0.0002 au with 5 × 10^3^ walkers is sufficient to keep statistical errors below 0.05 m*E*_h_, ensuring submilli-Hartree accuracy. However, for the final production calculations, we adopt a more conservative approach by doubling the number of walkers and reducing the time step by half. This ensures higher precision while further mitigating any residual time step or population-control bias.

In the production stage, we typically initialize the DMC simulations with 10^4^ walkers, obtained from the VMC simulation. These walkers are then propagated in imaginary time using a refined time step of Δ*τ* = 0.0001 au. The DMC simulations consist of two stages: equilibration and production. During equilibration (2 × 10^5^ DMC steps), the walker distribution is adjusted to better approximate the true ground state, allowing energy fluctuations to stabilize. In the subsequent production stage, approximately 1 × 10^6^ – 5 × 10^6^ DMC steps are performed to compute the final averaged DMC energy.

Additionally, to ensure a precise comparison between DMC and mean-field results, we performed UHF calculations using the largest available correlation-consistent basis sets. The molecular UHF calculations used to construct the PECs were performed using the aug-cc-pV6Z basis set for H_2_ and the aug-cc-pV5Z basis set for LiH and Li_2_ at the same internuclear separations as those used in the DMC calculations. The total UHF energies (*E*_HF_^tot^) obtained from these calculations serve as the reference for extracting the correlation energy at each bond distance. All calculations are performed using the GAUSSIAN09 package,^42^ with tight convergence criteria on both the electronic wave function and total energy.

All interaction energies reported below are measured relative to the corresponding separated atoms. Thus, for a diatomic molecule AB,

$$E_{\mathrm{HF}}\;\left(R\right)=E_{\mathrm{UHF}}^{\mathrm{tot}}\;\left(\mathrm{AB};R\right)-E_{\mathrm{HF}}^{\left(a\right)}\;\left(A\right)-E_{\mathrm{HF}}^{\left(a\right)}\;\left(B\right)$$

Following the UHF-based definition adopted here, the correlation contribution to molecular formation is obtained from the difference between the total and UHF interaction energies. The corresponding physical reference is therefore provided by the best available UHF energies of the separated ground-state atoms. For H, the atomic reference is *E*_HF_^(*a*)^(H) = −0.500000 *E*_*h*_. For Li, we use the CBS-extrapolated UHF energy of neutral Li in its 1*s* ^2^ 2*s* ^2^ *S* ground state, *E*_UHF_^(*a*)^(Li) = −7.432774 *E*_*h*_, reported in ref^39^. The LiH and Li_2_ molecular UHF energies were calculated using the aug-cc-pV5Z basis set; the resulting PECs are therefore CBS-referenced, near-CBS molecular curves. Explicit large-distance checks give residual HF interaction energies of +0.0334 m*E*_*h*_ for LiH at *R* = 9.0 Å and +0.0564 m*E*_*h*_ for Li_2_ at *R* = 10.0 Å. These results demonstrate that the molecular curves approach the CBS separated-atom limit to within 0.06 m*E*_*h*_, although they are not required to be exactly zero at these finite separations. Any constant energy shift arising solely from the chosen atomic reference does not affect the corresponding reaction forces, which are obtained from energy derivatives.

The use of UHF allows for partial inclusion of static correlation effects by permitting the independent optimization of spin-up and spin-down orbitals. This provides a more flexible reference energy compared to restricted Hartree–Fock, particularly in stretched molecular configurations, where near-degeneracies emerge. By employing the largest available basis sets and ensuring methodological consistency with DMC, we minimized basis set truncation effects and obtained a robust estimation of the correlation energy across the entire potential energy curve.

## III Results and Discussion

To isolate the interatomic interaction energy, we subtract the total energies of the constituent atoms. Since the studied molecules (H_2_, LiH, Li_2_) comprise only hydrogen and lithium atoms, we first define their atomic contributions. The hydrogen atom is a single-electron system with no correlation effects. Its exact ground-state energy is *E*_GS_^(*a*)^(H) = −0.50 *E*_h_. For the lithium atom (a three-electron system), the best ground-state energy is given by *E*_GS_^(*a*)^(Li) = −7.478060 *E*_h_, and the corresponding energy obtained in unrestricted Hartree–Fock is *E*_HF_^(*a*)^(Li) = −7.432774 *E*_h_.^39,51^ The atomic correlation energy for lithium is thus: *E*_c_^(*a*)^(Li) = *E*_GS_^(*a*)^(Li) – *E*_HF_^(*a*)^(Li) = −0.045286 *E*_h_.

For a diatomic system consisting of atoms A and B with internuclear distance (i.e., the bond distance) *R*, the interatomic interaction energy, including correlation effects, is obtained from DMC energies

2 $$E_{\mathrm{DMC}}=E_{\mathrm{DMC}}^{\mathrm{tot}}-\left(E_{\mathrm{GS}}^{\left(a\right)}\;\left(A\right)+E_{\mathrm{GS}}^{\left(a\right)}\;\left(B\right)\right)$$

Similarly, the interaction energy without electronic correlation is derived from the UHF total energies

3 $$E_{\mathrm{HF}}=E_{\mathrm{HF}}^{\mathrm{tot}}-\left(E_{\mathrm{HF}}^{\left(a\right)}\;\left(A\right)+E_{\mathrm{HF}}^{\left(a\right)}\;\left(B\right)\right)$$

The correlation contribution due to the interatomic interaction is then

4 $$E_{c}=E_{\mathrm{DMC}}-E_{\mathrm{HF}}$$

This approach reflects the standard definition of interaction energy: the difference between the total energy of the interacting atomic system and the sum of energies of the isolated atoms. Both QMC and UHF methods are employed to compute these energies (*E*_DMC_^tot^ and *E*_HF_^tot^), with their difference defining the total correlation energy (*E*_c_^tot^). The obtained interaction energies as a function of the bond distance produce the potential energy curves.

Finally, a key quantity for analyzing chemical processes is the reaction force.^27,52–54^ It can be obtained from the derivative of the total ground-state energy *E*_GS_ with respect to the internuclear distance *R*

5 $$F_{\mathrm{GS}}=-\frac{dE_{\mathrm{GS}}}{dR}$$

This force is zero at equilibrium (*F*_GS_ = 0 when *R* = *R*_*e*_) and exhibits extrema at inflection points of *E*_GS_, where the second derivative satisfies

6 $$\frac{d^{2}E_{\mathrm{GS}}}{dR^{2}}=-\frac{dF_{\mathrm{GS}}}{dR}=0$$

The value of the second derivative at the equilibrium bond distance *R* = *R*_*e*_ defines the bond force constant, or harmonic force constant.

In the present study, we approximate the ground-state energy of the diatomic system by the total DMC ground-state energy *E*_GS_ = *E*_DMC_^tot^. We define the corresponding interatomic forces as *F*_DMC_ = −d*E*_DMC_^tot^/*dR*, *F*_HF_ = −d*E*_HF_^tot^/d*R*, and *F*_c_ = −d*E*_c_^tot^/d*R*, with *F*_DMC_ = *F*_HF_ + *F*_c_.

### III.I H_2_ Molecule

For the two-electron H_2_ system, we perform DMC and UHF calculations across varying bond distances to obtain total energies *E*_DMC_^tot^ and *E*_HF_^tot^. The interaction energies relative to the total energy of two isolated hydrogen atoms are defined as *E*_DMC_ = *E*_DMC_^tot^ – 1.0*E*_h_ (with correlation) and *E*_HF_ = *E*_HF_^tot^ – 1.0*E*_h_ (without correlation), yielding the correlation energy contribution: *E*_c_ = *E*_DMC_^tot^ – *E*_HF_^tot^. Figure 1(a) displays the potential energy curves (PECs) for *E*_DMC_, *E*_HF_, and *E*_c_ in H_2_ as a function of the bond distance *R*. This work builds on pioneering studies of bond-distance-dependent correlation energies, including the full configuration interaction (CI) analysis of H_2_ by Rassolov, Ratner, and Pople (RRP),^32^ and later investigations by Wetthasinghe and Rassolov on LiH^+^ and LiH.^33^ The correlation energies for H_2_ obtained from the differences of the DMC and UHF energies show excellent agreement with reference data (*E*_c_^(RRP)^) taken from the RRP results in ref^32^. Our calculations for H_2_ provide a complete picture of electron correlation’s role throughout the bond dissociation process.

**Figure 1:**
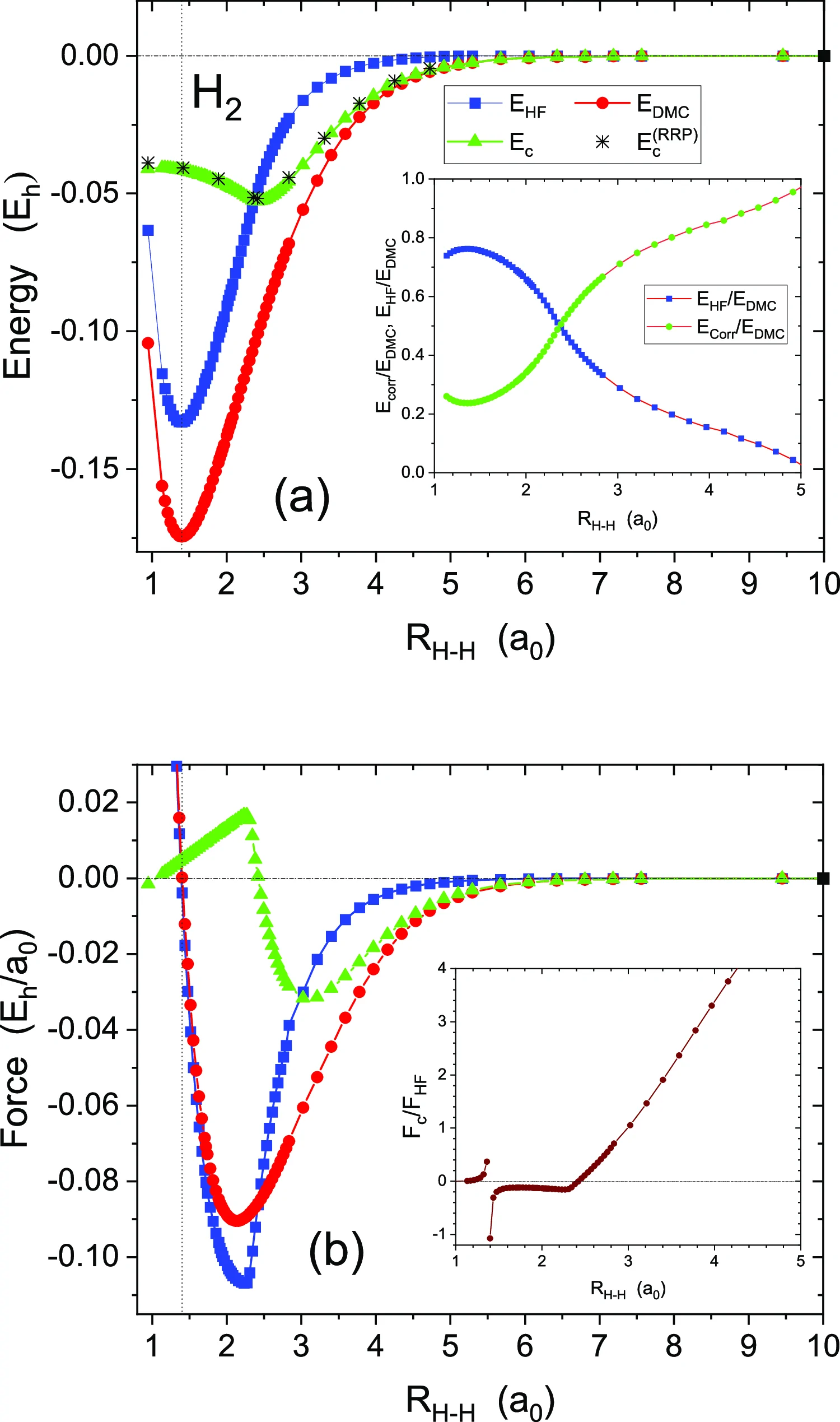
(a) Potential energy curves of the diatomic molecule H_2_ for the DMC (red dots), HF (blue squares), and correlation energies (green triangles). The correlation energy *E*_c_^(RRP)^ is taken from ref^32^. The inset shows the ratios *E*_HF_/*E*_DMC_ and *E*_c_/*E*_DMC_. (b) The corresponding interatomic forces are *F*_DMC_, *F*_HF_, and *F*_c_. The inset shows the ratio *F*_c_/*F*_HF_.

At the equilibrium bond distance *R*_*e*_ = 1.40*a*_0_, the final UHF/aug-cc-pV6Z calculation gives *E*_UHF_^tot^(H_2_) = −1.1336265 *E*_*h*_, corresponding to a Hartree–Fock interaction energy *E*_HF_ = −0.1336 *E*_*h*_ relative to two separated H atoms. The corresponding DMC total energy is *E*_DMC_^tot^(H_2_) = −1.1744665 *E*_*h*_, giving a total binding energy *E*_DMC_ = −0.1745 *E*_*h*_ (−4.7484 eV). Thus, the Hartree–Fock contribution accounts for 76.6% of the binding energy, while the correlation contribution is *E*_*c*_ = −0.0408 *E*_*h*_, or 23.4%. This decomposition confirms that while mean-field effects dominate near equilibrium, correlation provides a significant stabilization energy that cannot be neglected in accurate bonding descriptions. For H_2_ beyond *R* ≃ 2.25 *a*_0_, the UHF solution undergoes spin-symmetry breaking. In this region, UHF partially incorporates static correlation, and the residual DMC–UHF difference thus represents primarily dynamical correlation. This distinction is acknowledged in interpreting the long-range correlation tail.

We are particularly interested in the role of electron correlation in the formation of diatomic molecules. Figure 1(a) shows that for large *R*, *E*_HF_ approaches 0 much faster than *E*_DMC_ as *R* → ∞. This is evident in the inset, where the ratios *E*_HF_/*E*_DMC_ and *E*_c_/*E*_DMC_ are given as a function of the bond distance. We see that *E*_c_/*E*_DMC_ is larger than *E*_HF_/*E*_DMC_ for *R* > 2.37 *a*_0_. Quantitatively, *E*_c_ = *E*_HF_ = −0.0526 *E*_h_ at *R* = 2.37 *a*_0_. This crossover point marks the transition between HF-dominated covalent bonding and correlation-dominated weak interaction. At *R* = 5 *a*_0_, the correlation contribution *E*_c_ = −0.0038 *E*_h_ accounts for nearly all of the remaining interaction, while the HF contribution *E*_HF_ becomes negligible. Therefore, for *R* > 5 *a*_0_, *E*_c_ ≃ *E*_DMC_ as *E*_HF_ vanishes exponentially, leaving weak interatomic correlation as the sole contributor in the ground-state energy.

This confirms that for large *R* values, the weak interatomic interaction between two neutral atoms is purely due to correlation. It is known that the *R*-dependence of the correlation energy *E*_c_ is characteristic of multipole–multipole interactions at large distances. Such a correlation interaction is the origin of the London-dispersion force. In the elementary description, dispersion is often introduced as a long-range attraction between neutral fragments that arises from correlated charge fluctuations. In a stricter sense, dispersion is defined within long-range perturbation theory, whereas dynamic electron correlation is a broader concept that may also occur within a molecule or even within a single atom. Therefore, the correlation-induced attraction observed here should not be identified with dispersion in all respects; rather, it plays a dispersion-like or analogous physical role at large separations.

We note that, in their highly accurate calculations for H_2_, Kołos and Wolniewicz analyzed the energy splitting between the ground *X* ^1^Σ_*g*_^+^ state and the lowest triplet *b* ^3^Σ_*u*_^+^ state. This singlet–triplet splitting is associated with electron exchange symmetry and interatomic electron delocalization, and it decreases rapidly with increasing internuclear separation.^31^ Their results showed that this splitting is 0.005074 *E*_h_ at *R* = 5 *a*_0_ and only 0.000033 *E*_h_ at *R* = 8 *a*_0_. Our study is consistent with this behavior, but focuses instead on the UHF-reference correlation contribution within the ground *X* ^1^Σ_*g*_^+^ state.

The most striking finding emerges from analysis of the interatomic forces. The interatomic forces (reaction forces) are obtained by fitting to the numerical derivatives of the total DMC, HF, and correlation energies of the diatomic system. Figure 1(b) presents the interatomic forces *F*_DMC_ = −d*E*_DMC_^tot^/d*R*, *F*_HF_ = −d*E*_HF_^tot^/d*R*, and *F*_c_ = −d*E*_c_^tot^/d*R*, where forces *F*_HF_ and *F*_c_ arise from the interatomic HF and correlation interactions, respectively, and the total reaction force *F*_DMC_ = *F*_HF_ + *F*_c_. The total reaction force shows expected behavior, attractive for *R* > *R*_*e*_ and repulsive for *R* < *R*_*e*_. For *R* < *R*_*e*_, the magnitude of the repulsion force increases rapidly with decreasing *R* because of ionic core electrostatic repulsion. We observe that around the bond equilibrium, the HF component *F*_HF_ of the reaction force has characteristics similar to the total reaction force, but the component *F*_c_ reveals surprising complexity. Near equilibrium (1.06 *a*_0_ < *R* < 2.42 *a*_0_), *F*_c_ is actually repulsive, peaking at +0.017 *E*_h_/*a*_0_ at *R* = 2.25 *a*_0_. A related observation was reported by McDonagh, Vincent, and Popelier using MP2-IQA energy partitioning,^55^ where the interatomic correlation contribution in H_2_ was found to be positive, i.e., destabilizing. This supports the view that, in the covalent bonding region, the correlation contribution is not necessarily attractive. The present force decomposition extends this conclusion by showing explicitly that this destabilizing correlation contribution manifests as a repulsive component of the reaction force near the H_2_ equilibrium geometry. At the DMC equilibrium distance, the total force vanishes by definition, *F*_DMC_(*R*_*e*_) = 0. Consequently, the decomposed components satisfy *F*_HF_(*R*_*e*_) + *F*_*c*_(*R*_*e*_) = 0. The relevant information provided by the decomposition is not this cancellation itself, but the sign and magnitude of the individual components. In H_2_, *F*_*c*_ is repulsive near the equilibrium region and counteracts the attractive Hartree–Fock force. In general, the HF attraction force is much stronger within this range of bond distance. It underscores the dominant role of the HF interaction in midrange bonding. The combination of the HF and correlation interaction yields the maximum total attractive force, *F*_DMC_^max^ = −0.0904 *E*_h_/*a*_0_ at *R* = 2.12 *a*_0_. This point corresponds to an inflection point of the DMC potential energy curve, where d^2^ *E*_DMC_/d*R* ^2^= 0. Notice that, because of positive *F*_c_, the attractive force *F*_HF_^max^ of the HF component appears larger than *F*_DMC_^max^.

The sharp feature in the HF and correlation-force contributions slightly beyond the DMC inflection point is associated with the Coulson–Fischer symmetry-breaking region of the UHF solution. Below this region, the UHF solution remains equivalent to the spin-symmetric RHF solution, whereas beyond it, the opposite-spin orbitals become progressively localized on different atoms. Although the optimized UHF energy remains continuous, its curvature changes sharply, producing a kink in the HF force. Because *F*_c_ = *F*_DMC_–*F*_HF_ and the DMC force remains smooth, a compensating feature appears in the correlation-force contribution. This behavior reflects the decomposition relative to the broken-symmetry UHF reference rather than a physical discontinuity in the total molecular force.

For larger bond distance *R* > 2.42 *a*_0_, *F*_c_ is negative and reaches maximum attraction (−0.0316 *E*_h_/*a*_0_) at *R* = 3.02 *a*_0_ before weakening. In the inset in Figure 1(b), we plot the ratio *F*_c_/*F*_HF_, showing clearly that, for *R* > 3.0 *a*_0_, the ratio *F*_c_/*F*_HF_ > 1 and increases rapidly with increasing *R*. This happens because the HF attraction decreases exponentially at large *R*, but the Coulomb correlation carries the long-range nature of the Coulomb potential. Notice that a positive value of the ratio *F*_c_/*F*_HF_ means the two forces have the same sign and both are attractive in this case. The above results show clearly that, at a large bond distance, the weak attractive force is dominated by the dispersion contribution contained in the residual correlation energy.

In summary, the above calculations show quantitatively that electron correlation governs weak interatomic forces at dissociation of the covalent bond, while Hartree–Fock interactions dominate near equilibrium. The interplay between these components shapes the reaction force profile, with correlation counteracting HF attraction at *R*_*e*_. These results provide a force-level view of how the HF and residual correlation contributions evolve along the H_2_ dissociation coordinate. Along the dissociation coordinate, the weak long-range attraction between two far-separated neutral hydrogen atoms is dominated by dispersion, because the Hartree–Fock interaction decays rapidly at large *R*. With further decreasing *R*, the electron correlation *induces* the HF interaction. For the singlet ground state of H_2_, the HF contribution reflects the usual covalent mean-field stabilization associated with orbital delocalization and the redistribution of electronic density in the internuclear region. This should not be interpreted as an exchange interaction between opposite-spin electrons, since Hartree–Fock exchange acts only between same-spin electrons. Triplet states have different spin and spatial symmetry and are not considered in the present ground-state analysis. Each hydrogen atom has only one electron in the so-called open valence orbital; therefore, the electrons of different spins can penetrate (a quantum tunneling effect) between the atoms, leading to delocalization. At *R* = 4.5 *a*_0_, the HF interaction energy *E*_HF_ is about 10% of the total interaction energy *E*_DMC_. The exchange interaction between two H atoms in this situation is attractive, and the strength of the effective force *F*_HF_ increases rapidly with decreasing *R*. For *R* ≲3.0 *a*_0_, the ratio *F*_c_/*F*_HF_ is less than 1 (see the inset in Figure 1(b)) and the HF force *F*_HF_ becomes the dominant attractive force. As *R* decreases, the overlap between the atomic orbitals increases and the Hartree–Fock contribution grows rapidly, eventually becoming the dominant attractive component. This growth reflects the increasing energetic importance of the bonding combination and the redistribution of electronic density in the internuclear region. In the short-distance region, the UHF solution approaches the usual delocalized closed-shell description of the singlet ground state, whereas at stretched geometries, it may become broken-symmetry and more localized. Thus, the evolution should be understood in terms of increasing orbital overlap and changes in the UHF reference, rather than as the literal formation of a molecular orbital at a particular distance. Observing the correlation energy and the associated effective force we see that, with further reducing the bond distance *R*, though the correlation energy reaches its minimum value of −0.0528 *E*_h_ (maximum correlation) at *R* = 2.42 *a*_0_ (see Figure 1(a)), the interatomic force due to correlation already reaches its maximum attractive strength *F*_c_ = −0.0316 *E*_h_/*a*_0_ at *R* = 3.02 *a*_0_ (see Figure 1(b)). Then, *F*_c_ reduces its strength of attraction with decreasing *R* and becomes repulsive for 2.42 *a*_0_ > *R* > 1.06 *a*_0_. Notice that HF interactions dominate below 3.0 *a*_0_, driving bond formation via molecular orbital occupation.

### III.II LiH Molecule

Turning to the polar molecule LiH, we observe modified correlation effects due to the presence of the Li 1s core electrons and a more diffuse 2s valence orbital. In the Li-containing systems, the reported Hartree–Fock interaction energies use the aug-cc-pV5Z molecular UHF energies together with the CBS-extrapolated atomic Li HF reference defined in Sec. 2. Remember that, as given above, the ground-state energy of the Li atom is *E*_GS_^(*a*)^(Li) = −7.478060 *E*_h_. Within the UHF approximation, this energy is *E*_HF_^(*a*)^(Li) = −7.432774 *E*_h_. Therefore, the interaction energies for LiH are given by

$$E_{\mathrm{DMC}}=E_{\mathrm{DMC}}^{\mathrm{tot}}-E_{\mathrm{GS}}^{\left(a\right)}\;\left(\mathrm{Li}\right)-E_{\mathrm{GS}}^{\left(a\right)}\;\left(H\right)=E_{\mathrm{DMC}}^{\mathrm{tot}}+7.978060\;E_{h}$$

and

$$E_{\mathrm{HF}}=E_{\mathrm{HF}}^{\mathrm{tot}}-E_{\mathrm{HF}}^{\left(a\right)}\;\left(\mathrm{Li}\right)-E_{\mathrm{GS}}^{\left(a\right)}\;\left(H\right)=E_{\mathrm{HF}}^{\mathrm{tot}}+7.932774\;E_{h}$$

where the total energy *E*_DMC_^tot^ and *E*_UHF_^tot^ for the diatomic system are computed as functions of bond distance *R*.

Figure 2(a) displays the interaction energies *E*_DMC_, *E*_HF_, and *E*_c_ for LiH. The obtained correlation energies agree excellently with Wetthasinghe and Rassolov’s benchmark data (*E*_c_^W*R*^) obtained via full CI calculations.^33^ At equilibrium (*R*_*e*_ = 3.02 *a*_0_), *E*_DMC_ reaches its minimum −0.0921 *E*_h_(= −2.506 eV), corresponding to LiH’s binding energy. Here, Hartree–Fock and correlation contributions are *E*_HF_ = −0.0546 *E*_h_ (59%) and *E*_c_ = −0.0375 *E*_h_ (41%), respectively. Compared to H_2_, LiH exhibits a larger equilibrium bond distance (3.02 vs 1.40*a*_0_), a smaller binding energy (−2.5 vs −4.7 eV), and a greater correlation contribution (41% vs 24%). LiH differs electronically from H_2_ through the presence of the diffuse Li 2*s* valence orbital and the filled Li 1*s* core. The correlation contribution to the LiH PEC exhibits a broad plateau and a shallow nonmonotonic variation in this region: with increasing internuclear distance, its absolute magnitude first decreases and subsequently increases. The present calculations establish this *R*-dependent behavior but do not provide an orbital-resolved or core–valence-resolved decomposition from which its microscopic origin could be determined unambiguously.

**Figure 2:**
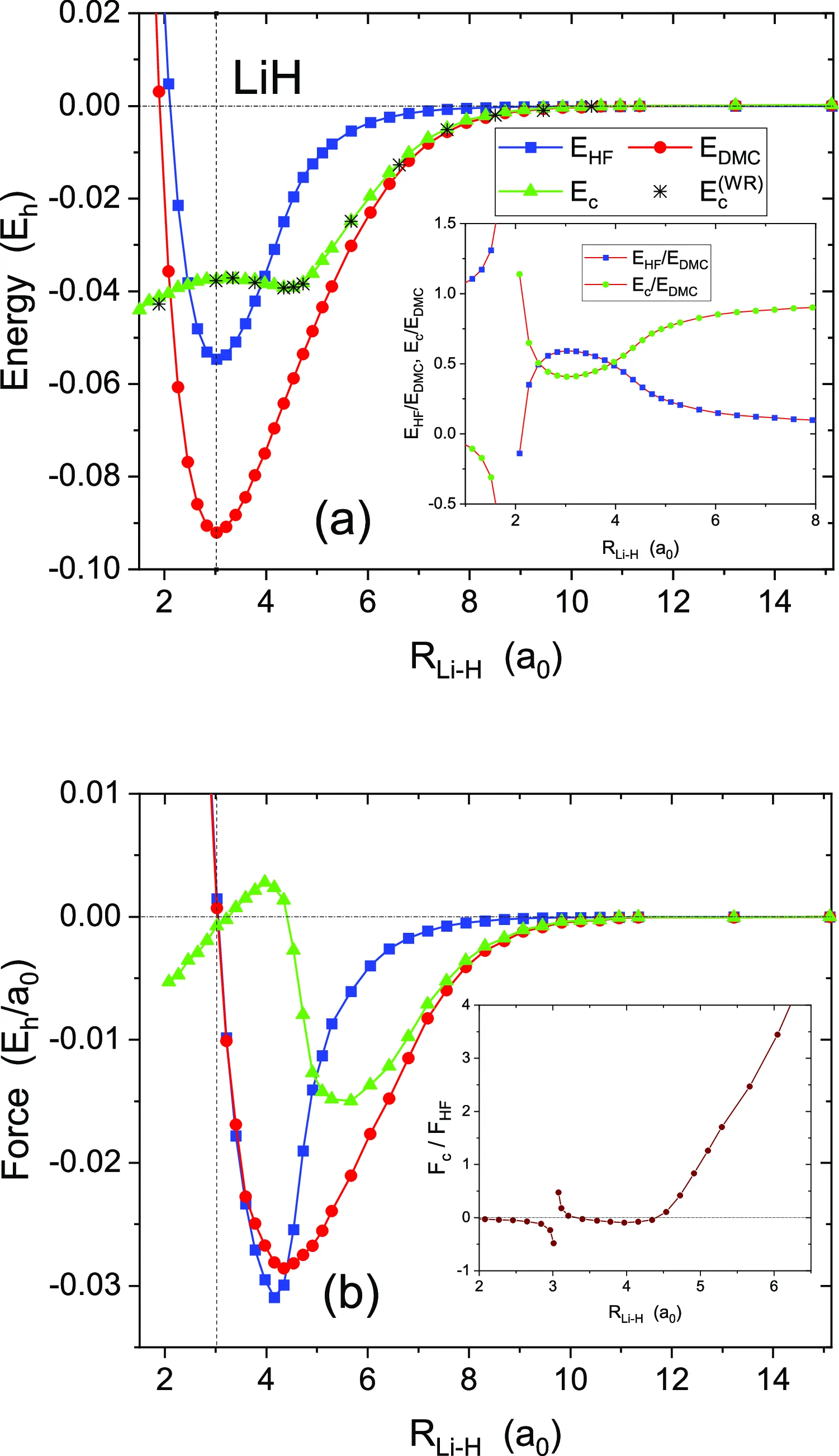
(a) Potential energy curves of the diatomic molecule LiH for *E*_DMC_ (red dots), *E*_HF_ (blue squares), and *E*_c_ (green triangles). The correlation energy *E*_c_^(W*R*)^ is taken from ref^33^. The inset shows the ratios *E*_HF_/*E*_DMC_ and *E*_c_/*E*_DMC_. (b) The corresponding interaction forces are *F*_DMC_, *F*_HF_, and *F*_c_. The inset shows the ratio *F*_c_/*F*_HF_.

Although the HF energy *E*_HF_ is dominant around the equilibrium bond distance, as shown in the figure, the correlation energy *E*_c_ in the LiH system is significantly more important for larger *R*. The inset in Figure 2(a) shows the ratios *E*_HF_/*E*_DMC_ and *E*_c_/*E*_DMC_. It is seen that *E*_HF_/*E*_DMC_ is larger than *E*_c_/*E*_DMC_ for 2.47 < *R* < 3.91 *a*_0_. However, the correlation energy *E*_c_ becomes dominant for *R* > 3.91 *a*_0_, and is about 10 times larger than *E*_HF_ at *R* ≃ 8 *a*_0_. The crossover point where |*E*_c_| = |*E*_HF_| occurs at *R* > 3.91 *a*_0_, significantly larger than in H_2_ due to the more diffuse orbitals. Concretely, *E*_c_ = *E*_HF_ = −0.0382 *E*_h_ at *R* = 3.91 *a*_0_. And *E*_c_ = −0.0031 *E*_h_ (≫ *E*_HF_) at *R* = 8 *a*_0_.

Figure 2(b) plots the total reaction force *F*_DMC_ = −d*E*_DMC_/d*R*, alongside HF and correlation components *F*_HF_ and *F*_c_, showing the reaction-force analysis. The interatomic forces are obtained by the best fitting to the numerical derivatives of the interaction energies with respect to the interatomic distance. The force analysis reveals behavior distinct from H_2_. It is seen that *F*_c_ due to electron correlation is dominant in the large bond distance for *R* ≳ 5.0 *a*_0_, where *F*_c_/*F*_HF_ > 1 as shown in the inset in Figure 2(b), reflecting the Coulomb correlation as the sole driver of weak interatomic attraction force (the dispersion force) for larger *R*. When *R* decreases from 5.0 *a*_0_, the HF attraction force *F*_HF_ is enhanced rapidly while the strength of *F*_c_ decreases. In the midrange transition (3.2 *a*_0_ < *R* < 4.4 *a*_0_), *F*_c_ is repulsive (positive), peaking at +0.017 *E*_h_/*a*_0_ (*R* = 4.4 *a*_0_). At the equilibrium bond distance *R*_*e*_ = 3.02 *a*_0_, *F*_c_ = −0.0007 *E*_h_/*a*_0_ is slightly attractive, but *F*_HF_ = +0.0007 *E*_h_/*a*_0_ is repulsive, ensuing total reaction force *F*_DMC_ = 0.

The above investigation for the LiH molecule shows that, at large interatomic separation, weak correlation-driven attraction initiates bond formation. With decreasing the bond distance *R*, the correlation induces the HF attraction between Li and H atoms. Because the two atoms have open valence orbitals with only one electron each, electron tunneling or penetration between Li­(2*s*) and H­(1*s*) orbitals enhances the HF attraction in the intermediate stage rapidly (*R* ≲ 5.0 *a*_0_). When the total attraction force reaches its maximum strength *F*_DMC_^max^ = −0.0286 *E*_h_/*a*_0_ at *R* = 4.35 *a*_0_, the total energy of the system is at its turning point with d^2^ *E*_DMC_/d*R* ^2^ = −d*F*_DMC_/d*R* = 0. With further decreasing of the distance between the atoms from this turning point, the total attraction force reduces its strength and the total energy of the two interacting atoms reaches its minimum value. The interplay of the HF and correlation interactions stabilizes the LiH molecule to its equilibrium ground state, forming a stable molecular bond. Notably, at *R*_*e*_, correlation introduces a slight attraction (*F*_c_ < 0), in contrast to the behavior in H_2_.

### III.III Li_2_ Molecule

For the diatomic molecule Li_2_, the interatomic interaction energy with full electron correlation is defined as *E*_DMC_ = *E*_DMC_^tot^ – 2 *E*_GS_^(*a*)^(Li) = *E*_DMC_^tot^ + 14.95612 *E*_h_ and the HF interaction energy is obtained by *E*_HF_ = *E*_HF_^tot^ – 2 *E*_HF_^(*a*)^(Li) = *E*_UHF_^tot^ + 14.865548 *E*_h_. The correlation contribution to the interaction energy is given by *E*_c_ = *E*_DMC_ – *E*_HF_. They are plotted as a function of bond distance in Figure 3(a).

**Figure 3:**
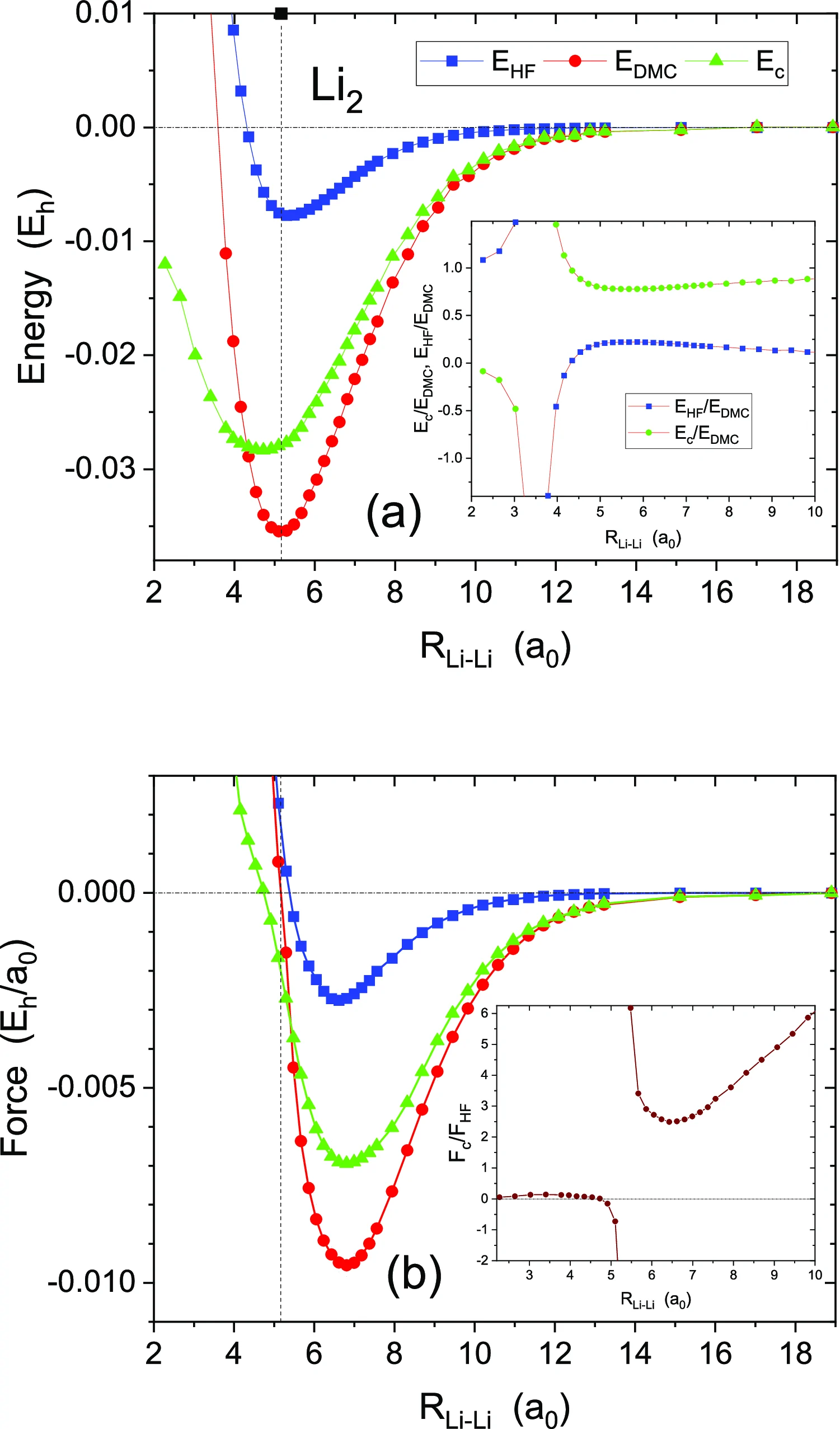
(a) Potential energy curves of the diatomic molecule Li_2_ for *E*_DMC_ (red dots), *E*_HF_ (blue squares), and *E*_c_ (green triangles). The inset shows the ratios *E*_HF_/*E*_DMC_ and *E*_c_/*E*_DMC_. (b) The corresponding interaction forces are *F*_DMC_, *F*_HF_, and *F*_c_. The inset shows the ratio *F*_c_/*F*_HF_.

The Li_2_ molecule presents the most dramatic case of correlation-dominated bonding. The DMC calculation shows the maximum interaction energy *E*_DMC_ = −0.0354 *E*_h_ (−0.963 eV) at equilibrium *R*_*e*_ = 5.16 *a*_0_. This is the binding energy of the Li_2_ molecule with a remarkable correlation contribution 79% (*E*_c_ = −0.0279 *E*_h_) of the total binding energy and *E*_HF_ accounting for just 21% (*E*_HF_ = −0.0075 *E*_h_). Notably, *E*_c_ is almost four times larger than *E*_HF_ in the composition of the binding energy of the Li_2_ molecule. This behavior reflects the diffuse character of the Li 2*s* valence orbital, the screening and Pauli repulsion associated with the Li 1*s* core electrons, and the absence of electronegativity-driven polar or ionic stabilization in the homonuclear Li–Li bond. In fact, in the whole range of the bond distance under investigation, the correlation dominates the interaction energy, as shown in the inset in Figure 3(a). Notice that *E*_DMC_ = 0 at *R* = 3.58 *a*_0_, where *E*_HF_/*E*_DMC_ diverges as shown in the inset. For a smaller *R*, *E*_DMC_ > 0, indicating that two atoms strongly repel each other. The maximum relative HF contribution measured by the ratio *E*_HF_/*E*_DMC_ is only about 22% (at *R* = 5.9 *a*_0_). When the two Li atoms are apart from each other, *E*_HF_ decreases rapidly, approaching zero. Similarly to H_2_ and LiH, electron correlation drives a weak attraction between two widely separated neutral Li atoms. In this case, part of the long-range attraction likely originates from correlations involving the closed-shell 1*s* cores, which become increasingly significant for larger atoms or clusters.

Figure 3(b) shows that the maximum attractive reaction force occurs at *R* = 6.80 *a*_0_ with *F*_DMC_^max^ = −0.0095 *E*_h_/*a*_0_. The interaction energy and reaction force in the Li–Li bond, as shown in Figure 3, are much smaller than those in the H–H bond in H_2_ and the Li–H bond in LiH molecule. Weak bonding of the Li_2_ molecule is mostly related to the large Li atomic radius (*r*_Li_ = 3.27 *a*_0_ determined by the 2*s* orbital), exchange repulsion, and screening of the 1*s* core electrons. However, the correlation contribution in Li_2_ is significant. Both the interatomic interaction energy and reaction force are dominated by the electron correlation in the Li_2_ diatomic system from far-separated atoms to the equilibrium molecule. We also find that the effective force *F*_c_ due to correlation is attractive at equilibrium *R*_*e*_ = 5.16 *a*_0_ with *F*_c_ = −0.0018 *E*_h_/*a*_0_ = −F_HF_, as shown in Figure 3(b). Notice that *F*_HF_ = 0 at *R* = 5.4 *a*_0_ > *R*_*e*_ = 5.16 *a*_0_, where the ratio *F*_c_/*F*_HF_ diverges and changes sign around this bond distance, as shown in the inset. In fact, |*F*_c_/*F*_HF_| > 1 for *R* > *R*_*e*_, indicating that the total reaction force is dominated by the correlation effect for large *R*. In Li_2_, correlation effects dominate the interaction energy, contributing nearly 80% of the binding at equilibrium, which illustrates how correlation-driven attraction bridges the dispersion and covalent regimes.

### III.IV Unified View of Electron Correlation in Chemical Bonding

Our analysis of the three prototype diatomic systems (H_2_, LiH, and Li_2_) shows how electron correlation contributes across different bonding regimes. Although these examples cannot represent all chemical species, they illustrate in a quantitative way the continuous evolution from long-range dispersion to short-range covalent stabilization within a consistent quantum-mechanical framework. In this sense, the results exemplify—rather than attempt to generalize—how correlation operates across bonding types. It is useful to relate this decomposition to more traditional bonding interpretations. The Hartree–Fock part of the interaction contains the mean-field contributions associated with charge redistribution, electrostatic attraction, exchange, orbital delocalization, and interatomic electron motion. Thus, in H_2_ and LiH, where *E*_HF_ dominates near equilibrium, the main covalent stabilization remains closely connected to the Hellmann–Ruedenberg and Feynman-type pictures of bonding. The correlation term defined here should therefore not be interpreted as a replacement for these mechanisms. Rather, *E*_c_ = *E*_DMC_ – *E*_HF_ measures the additional many-electron stabilization beyond the UHF reference and shows how this residual correlation modifies the interaction energy and reaction force along the dissociation coordinate. At large separations, where the HF interaction decays rapidly, this residual correlation becomes the dominant attractive contribution and connects naturally with dispersion-like interactions.

The present results also provide a reference point for more commonly used electronic-structure methods. DMC is used here as a high-accuracy benchmark rather than as a routine replacement for standard quantum-chemical approaches. Near equilibrium, where the electronic structure is largely single-reference, methods such as coupled-cluster theory or suitably constructed KS-DFT functionals can recover much of the dynamical correlation. At stretched geometries, however, static or left–right correlation becomes important, and single-reference RHF descriptions suffer from the familiar dissociation error. UHF partially removes this error through spin-symmetry breaking, while spin-adapted valence-bond or CASSCF-type descriptions provide a more physical multiconfigurational reference. Such methods, however, still require additional treatment of dynamical correlation. From this perspective, the DMC–UHF difference analyzed here measures the residual correlation beyond a broken-symmetry mean-field reference and provides useful benchmarks for assessing how approximate methods treat both near-equilibrium correlation and the long-range dispersion-like tail.

The case of LiH also deserves comment in relation to the distinction between polar and ionic bonding. Within QCT/QTAIM analyses, charge transfer obtained from topological atomic charges provides a direct measure of ionicity in diatomic molecules, and LiH may therefore be regarded as having substantial ionic character.^17–20^ In the present work, we refer to LiH as a polar diatomic molecule to emphasize that its stabilization contains several contributions: charge-transfer/electrostatic effects, Hartree–Fock exchange interactions, and electron correlation. Thus, our results are consistent with a significant ionic component in LiH, while showing that correlation still provides a substantial part of the binding energy and modifies the force balance along the dissociation coordinate. Within this context, our calculations demonstrate that electron correlation can be viewed as a common physical mechanism connecting the limits of dispersion and covalency. At large interatomic separations, where HF interactions vanish, the only remaining attractive force originates from correlation effects, manifesting as London dispersion. This behavior—well established in the literature^27,56^ —arises from long-range Coulomb correlations between electrons in spatially separated atoms.

Fundamentally, electron correlation is a quantum mechanical phenomenon resulting from the interplay between the singular nature of the Coulomb interaction and the nonlocality of electron wave functions. The singularity in the relative coordinate **r**_*ab*_ = **r**_*a*_ – **r**_*b*_ produces a *Coulomb hole* around **r**_*ab*_ = 0, along with associated cusp conditions that constrain the electronic wave function.^13,57^ The strength of the correlation energy depends critically on wave function overlap: it is maximized when electrons occupy the same molecular orbital with finite joint density at the center of the Coulomb hole (**r**_*ab*_ = 0), and minimized when the orbitals are nonoverlapping, as in widely separated atoms. Notably, electrons are correlated through the long-range Coulomb potential, and the resulting correlation therefore retains a long-range character.

In the diatomic systems studied, this general behavior is translated into specific trends. At large distances, weak interatomic correlation drives dispersion forces in H_2_, LiH, and Li_2_, thereby initiating bond formation. As the fragments are brought closer along the dissociation coordinate, the HF contribution grows rapidly, reflecting the increasing importance of the mean-field charge redistribution and orbital delocalization in the bonding region. The residual correlation contribution then describes the remaining many-electron effects beyond this UHF reference, including short-range dynamical correlation near-equilibrium and dispersion at larger separations. This effect is most pronounced in Li_2_, where diffuse orbitals and weak exchange interactions allow the correlation to dominate (accounting for approximately 79% of the binding energy at equilibrium). Quantitatively, we observe that the correlation energy reaches a maximum at intermediate bond distances, slightly beyond equilibrium for all three systems. Specifically, the maximum values are −0.0528 *E*_h_ at *R* = 2.42 *a*_0_ for H_2_ (*R*_*e*_ = 1.40 *a*_0_), −0.0392 *E*_h_ at *R* = 4.40 *a*_0_ for LiH (*R*_*e*_ = 3.02 *a*_0_), and −0.0283 *E*_h_ at *R* = 4.72 *a*_0_ for Li_2_ (*R*_*e*_ = 5.16 *a*_0_). These maxima align with the distance where wave function overlap is sufficient to enhance intraorbital correlation, but before repulsion and kinetic constraints limit further stabilization.

The correlation-energy curves also suggest two physically distinct regimes, most clearly in H_2_ and LiH. In the outer regime, at large *R*, the Hartree–Fock interaction has largely vanished, and the remaining attraction is dominated by interfragment dynamical correlation. This is the dispersion-like regime, where *E*_c_ rises smoothly toward zero as the fragments separate. In the inner regime, near and inside the equilibrium region, *E*_c_ reflects residual intramolecular or intraorbital correlation in the presence of substantial orbital overlap. In H_2_ and LiH, this inner contribution is relatively flat because several effects partially compensate each other: increasing orbital overlap enhances correlation, whereas Pauli repulsion, kinetic constraints, UHF spin-symmetry breaking, and, in LiH, core screening and bond polarity limit further correlation stabilization. Since the force contribution *F*_c_ is determined by the slope of *E*_c_, this compensation naturally leads to weak or sign-changing correlation forces near equilibrium. Li_2_ behaves differently because the Hartree–Fock attraction is weak over the entire bonding region, while the diffuse Li 2s orbitals make correlation the dominant source of stabilization. Consequently, the inner and outer correlation regimes are less clearly separated, and *E*_c_ itself resembles a bond-energy curve.

A consistent physical picture emerges from these analyses. As the bond distance increases, the Hartree–Fock contribution decays exponentially while the correlation component retains its long-range character, governing the residual dispersion attraction. At shorter distances, the same correlation effects act cooperatively with exchange to stabilize the covalent bond. Across the three prototype systems: H_2_, LiH, and Li_2_, the relative weight of correlation increases systematically with atomic size and orbital diffuseness, while the crossover points where *E*_c_ = *E*_HF_ mark the transition between dispersion- and covalent-dominated regimes. These trends provide quantitative evidence that correlation acts as a connecting thread between different bonding regimes and set the stage for a broader interpretation.

## IV Conclusion

Our study provides a quantitative perspective on how electron correlation connects weak dispersion interactions to strong covalent bonding. By performing high-accuracy QMC and UHF calculations on H_2_, LiH, and Li_2_, we have shown that correlation is not merely a small correction to mean-field theories but a key component that strongly influences the character of bonding across different regimes. These systems were selected as representative prototypes that allow this evolution to be analyzed clearly and comparatively along their dissociation coordinates rather than to provide a general description of all chemical species.

At large interatomic separations, correlation accounts for essentially all of the interaction energy, manifesting as a dispersion-like attraction. As the atoms approach, correlation progressively modifies and stabilizes covalent bonding, contributing about 24% of H_2_’s binding energy at equilibrium and increasing to nearly 80% for Li_2_. Notably, a repulsive correlation force appears near the H_2_ equilibrium geometry, whereas in Li_2_, it remains attractive, reflecting underlying differences in orbital overlap and exchange interactions. These results demonstrate that correlation can both reinforce and counteract mean-field interactions, depending on the bonding regime.

The reaction-force analysis further clarifies how correlation affects bond formation. By decomposing the total force as *F*_DMC_ = *F*_HF_ + *F*_c_, we find that the correlation force provides the dominant long-range attraction in all three systems, consistent with the correlation-driven origin of dispersion-like interactions at large separations. Near equilibrium, however, the role of *F*_c_ is molecule-dependent. In H_2_, *F*_c_ becomes repulsive near *R*_*e*_, counteracting the Hartree–Fock attraction and helping to explain the tendency of HF theory to predict shorter bond lengths. In LiH, the correlation force is weak near equilibrium and changes sign across the bonding region, reflecting the competition between polar bonding, core screening, and valence-electron correlation. In Li_2_, *F*_c_ remains attractive at equilibrium and dominates the total restoring force over a broad range of bond distances, consistent with the correlation-dominated character of its weak bond.

By mapping correlation energies and reaction forces along the full dissociation coordinate, we identified crossover points where correlation and HF contributions became comparable, signaling the transition between dispersion-dominated and covalent regimes. This analysis also highlights the limitations of commonly used approximate density functionals, particularly in systems such as Li_2_, where correlation effects dominate.

Overall, the present results suggest a continuous physical connection between the long-range dispersion and short-range covalent stabilization. While demonstrated here for three prototypical diatomic systems, this Perspective offers a physically transparent framework for interpreting bonding in more complex systems and for improving electronic-structure methods that require a balanced treatment of exchange and correlation. Future extensions to multielectron and transition-metal systems will further test the robustness of this correlation-based interpretation.

## Supplementary Material



## Notes

Sharp feature in the HF and correlation-force contributions slightly beyond the DMC inflection point is associated with the Coulson–Fischer symmetry-breaking region of the UHF solution (PDF)
