# Supplementary material for: Electron Correlation in Chemical Bonding: From Dispersion to Covalent Stabilization in Prototype Diatomic Systems
Source: J Phys Chem A. 2026 Sep 25;130(40):7875–86. doi: 10.1021/acs.jpca.6c02311 (PMC13656773; doi:10.1021/acs.jpca.6c02311)
Supplement: Supplementary file 1 [file jp6c02311_si_001.pdf]

Supporting Information

Electron Correlation in Chemical Bonding: From Dispersion to Covalent Stabilization in Prototype Diatomic Systems

Guo-Qiang Hai[1], Ladir Cândido[2], and Braulio Gabriel Alencar Brito[3]

[1]Instituto de Física de São Carlos, Universidade de São Paulo, SP, Brazil

[2]Instituto de Física, Universidade Federal de Goiás, GO, Brazil

[3]Departamento de Física, Universidade Federal do Triângulo Mineiro, MG, Brazil

S1. Coulson–Fischer Feature in the H_2 Force Decomposition

The sharp feature in the Hartree–Fock and correlation-force contributions in Figure 1(b) of the main article occurs in the Coulson–Fischer symmetry-breaking region of the unrestricted Hartree–Fock (UHF) solution. At shorter internuclear distances, the lowest-energy UHF solution coincides with the spin-symmetric restricted Hartree–Fock (RHF) solution. Beyond the symmetry-breaking point, the spin-up and spin-down orbitals become progressively localized on different hydrogen atoms. This additional variational freedom allows UHF to approach the separated-atom limit and partially account for the static left–right correlation missing from RHF.[1]

The optimized UHF energy remains continuous through this region, but its curvature changes sharply. Since the reaction force is the negative derivative of the energy with respect to the internuclear distance R, this change in curvature produces a kink in the HF force. The force decomposition used in the main article is

$$E_c(R) = E_{\mathrm{DMC}}(R) - E_{\mathrm{HF}}(R), \qquad F_c(R) = F_{\mathrm{DMC}}(R) - F_{\mathrm{HF}}(R), \tag{S1}$$

where the energies are interaction energies relative to the corresponding separated atoms, and E_{HF} denotes the UHF reference. Because F_{DMC} is smooth across this region, the feature in F_{HF} appears with a compensating character in F_c. In terms of energy curvatures,

$$\frac{dF_c}{dR} = -\frac{d^2E_{\mathrm{DMC}}}{dR^2} + \frac{d^2E_{\mathrm{HF}}}{dR^2}. \tag{S2}$$

Thus, the sharp features in the two components cancel in their sum, $F_{\mathrm{DMC}} = F_{\mathrm{HF}} + F_c$. They reflect the change in the UHF reference and the resulting decomposition, rather than a physical discontinuity in the total DMC potential-energy or force curve.

S2. Atomic Reference Energies and Large-Distance Consistency Checks

S2.1. Interaction-energy convention

For a diatomic molecule AB, the HF interaction energy is defined as

$$E_{\mathrm{HF}}(R) = E_{\mathrm{UHF}}^{\mathrm{tot}}(\mathrm{AB}; R) - E_{\mathrm{HF}}^{(a)}(\mathrm{A}) - E_{\mathrm{HF}}^{(a)}(\mathrm{B}), \tag{S3}$$

where the superscript (a) denotes an isolated atomic reference. The correlation contribution to the interaction energy is given by eq (S1); it therefore excludes the correlation energies of the separated atoms.

The molecular UHF calculations employ aug-cc-pV6Z for H_2 and aug-cc-pV5Z for LiH and Li_2. The hydrogen atomic HF reference is $E_{\mathrm{HF}}^{(a)}(\mathrm{H}) = -0.500000\,E_{\mathrm{h}}$. For neutral lithium in its $1s^2 2s\,^2S$ ground state, the reference is the complete-basis-set (CBS) extrapolated UHF energy,[2]

$$E_{\mathrm{HF}}^{(a)}(\mathrm{Li}) = -7.432774\,E_{\mathrm{h}}. \tag{S4}$$

Accordingly, the LiH and Li_2 curves are large-basis molecular curves referred to CBS atomic energies. They are distinct from a strictly finite-basis supermolecular convention in which molecular and isolated-fragment energies are calculated with a common finite basis prescription. The numerical checks below assess the compatibility of the adopted atomic references with the large-distance molecular results.

S2.2. Atomic and molecular numerical checks

An independent aug-cc-pVQZ calculation for ground-state neutral Li gives

$$E_{\mathrm{UHF}}^{(a)}(\mathrm{Li}; \text{aug-cc-pVQZ}) = -7.432718717457\,E_{\mathrm{h}}, \tag{S5}$$

which is approximately $0.0553\,\mathrm{m}E_{\mathrm{h}}$ above the adopted CBS reference. Table S1 reports the molecular checks at large internuclear separations. The residual interaction energies are calculated directly from eq (S3) using the atomic references above.

Table S1: Large-distance UHF total energies and residual HF interaction energies for the aug-cc-pV5Z molecular calculations.

Molecule	R (Å)	$E_{\mathrm{UHF}}^{\mathrm{tot}}$ (E_{h})	E_{HF} (mE_{h})
LiH	9.0	−7.93274058631	+0.0334
Li_2	10.0	−14.8654915624	+0.0564

For example, the LiH residual follows from

$$\begin{aligned} E_{\mathrm{HF}}(\mathrm{LiH}; 9.0\,\text{Å}) &= \big[-7.93274058631 - (-7.432774 - 0.500000)\big]E_{\mathrm{h}} \\ &= +0.00003341369\,E_{\mathrm{h}} \simeq +0.0334\,\mathrm{m}E_{\mathrm{h}}. \end{aligned} \tag{S6}$$

Both molecular energies are within $0.06\,\mathrm{m}E_{\mathrm{h}}$ of the corresponding sums of the adopted atomic references at the separations tested.

S2.3. Consistency of the inferred Li fragment energies

A further consistency check compares effective Li fragment energies inferred independently from the two large-distance molecular calculations. Subtracting the H reference from the LiH total energy gives

$$\begin{aligned} E_{\text{Li,eff}}^{\text{LiH}} &= E_{\text{UHF}}^{\text{tot}}(\text{LiH}; 9.0\,\text{Å}) - E_{\text{HF}}^{(a)}(\text{H}) \\ &= -7.43274058631\,E_{\text{h}}. \end{aligned} \tag{S7}$$

Half of the Li_2 total energy gives

$$E_{\text{Li,eff}}^{\text{Li}_2} = \frac{1}{2} E_{\text{UHF}}^{\text{tot}}(\text{Li}_2; 10.0\,\text{Å}) = -7.43274578120\,E_{\text{h}}. \tag{S8}$$

These values differ by approximately $0.0052\,\text{m}E_{\text{h}}$. Their deviations from the adopted CBS atomic reference are summarized in Table S2. Because they are inferred at finite separations, they include any residual molecular interaction and should not be identified with independent isolated-atom calculations.

Table S2: Effective Li fragment energies inferred from large-distance molecular UHF results. The deviation is measured relative to $-7.432774\,E_{\text{h}}$.

Source	Effective Li energy (E_{h})	Deviation ($\text{m}E_{\text{h}}$)
LiH, $R = 9.0$ Å	−7.43274058631	+0.0334
Li_2, $R = 10.0$ Å	−7.43274578120	+0.0282

The agreement between the two inferred fragment energies supports the mutual consistency of the molecular calculations and their compatibility with the adopted atomic reference. The small positive residuals combine finite-basis and numerical effects with any remaining physical interaction at the finite distances considered; these checks do not separately determine those contributions or establish a rigorous basis-set error bound.

S2.4. Consequences for reaction forces

A constant change in an atomic reference shifts the interaction-energy curve by a constant and therefore does not change its derivative,

$$-\frac{d}{dR}[E_{\text{HF}}(R) + C] = -\frac{dE_{\text{HF}}(R)}{dR}, \qquad C = \text{constant}. \tag{S9}$$

Consequently, a constant reference shift changes the energy decomposition but not the HF force or the corresponding correlation force. A residual physical interaction at finite R, however, is generally R-dependent and is not a constant reference shift.

References

[1] Hollett, J. W.; Gill, P. M. W. The Two Faces of Static Correlation. *J. Chem. Phys.* **2011**, *134*, 114111.

[2] Hai, G.-Q.; Cândido, L.; Brito, B. G. A.; Liu, Y. Revealing the Regularities of Electron Correlation Energies Associated with Valence Electrons in Atoms in the First Three Rows of the Periodic Table. *Chem. Phys. Lett.* **2024**, *855*, 141567.
